# Cryoablation combined with transarterial infusion of pembrolizumab (CATAP) for liver metastases of melanoma: an ambispective, proof-of-concept cohort study

**DOI:** 10.1007/s00262-020-02566-z

**Published:** 2020-04-24

**Authors:** Lujun Shen, Han Qi, Shuanggang Chen, Fei Cao, Lin Xie, Ying Wu, Weimei Ma, Ze Song, Hui Yuan, Tao Zhang, Dandan Li, Xizhi Wen, Qifeng Chen, Wang Li, Xiaoshi Zhang, Weijun Fan

**Affiliations:** 1 Department of Minimally Invasive Interventional Therapy, Sun Yat-Sen University Cancer Center, Guangzhou, 510060 People’s Republic of China; 2 State Key Laboratory of Oncology in South China, Collaborative Innovation Center of Cancer Medicine, Sun Yat-Sen University, Guangzhou, 510060 People’s Republic of China; 3 Department of Biological Therapy Center, Sun Yat-Sen University Cancer Center, Guangzhou, 510060 People’s Republic of China; 4 Department of Medical Imaging, Seventh Affiliated Hospital of Sun Yat-Sen University, Shenzhen, 518107 People’s Republic of China; 5 Intelligence Technology Co. Ltd, Guangzhou, 510060 People’s Republic of China

**Keywords:** Melanoma, Liver metastasis, Cryoablation, Pembrolizumab, Proof-of-concept

## Abstract

**Background:**

The presence of liver metastasis correlates with poor therapeutic response of PD-1 blockade therapy in melanoma. A novel treatment protocol by combining cryoablation with transarterial infusion of pembrolizumab (CATAP) was proposed, and its feasibility and safety was assessed among this group of patients.

**Methods:**

This registered ambispective cohort study enrolled fifteen melanoma patients with multiple hepatic metastases who received planned two-stage CATAP therapy: in the combined stage, subtotal cryoablation on day 1, in which one to two intrahepatic lesions were ablated completely with other lesions left untreated, sequentially combined transarterial infusion of pembrolizumab on day 3, every three weeks, for at least one cycle; in the infusion stage, arterial infusion of pembrolizumab was recommended at three-week interval until disease progression. The primary endpoint was objective response rate by RECIST (version 1.1); secondary end points included progression-free survival (PFS) and safety; exploratory endpoints were changes of cytokines and immune cell compositions in peripheral blood samples.

**Results:**

Of the 15 patients enrolled, no grade 3–4 adverse events or major complications were observed. One patient (6.7%) achieved complete response, and 3 (20.0%) achieved partial response. The overall response rates of CATAP for the entire cohort and patients with cutaneous melanoma were 26.7% (95% confidence interval (CI) 4.3–49.0%) and 33.3% (95% CI 2.5–64.1%), respectively. Clinical response was observed in a proportion of patients (2/6; 33.3%) who failed first-line intravenous pembrolizumab treatment. The median overall PFS time and hepatic PFS time were 4.0 (95% CI 2.5–5.5) and 5.73 (95% CI 1.1–10.4) months, respectively. A significant increase in CD3-CD16 + CD56 + cells (natural killer cells; *P* = 0.0124) and a marginally significant decrease in CD4 + CD25 + cells (regulatory T cells; *P* = 0.0546) were observed three weeks after the first cycle of treatment in the combined stage.

**Conclusions:**

The CATAP therapy demonstrated positive clinical activity and a favorable safety profile for melanoma patients with liver metastasis.

## Introduction

With the advent of immune checkpoint inhibitors in recent years, significant progress is achieved in the management of advanced melanoma [1–3]. However, liver metastasis remains a clinical challenge and had been demonstrated as an independent predictive factor for reduced response and poor outcome in metastatic melanoma patients receiving intravenous PD-1 blockade immunotherapy. The median progression-free survival (PFS) times for patients with liver involvement receive intravenous PD-1 blockade therapy ranging from 2.7 to 5.1 months and for those without liver involvement ranging from 18.5 to 20.1 months [4].

Various mechanisms of local immune tolerance in the liver have been proposed, including the elevated expression of anti-inflammatory molecules such as IL-10 induced by pathogen-derived molecules [5], direct cell–cell contact between liver sinusoidal endothelial cells (LSECs) and liver DCs in the microenvironment [6], expansion of regulatory T cells and trapping and deletion of activated CD8 + cells [7]. The exact reason of the low response rate of anti-PD-1 monotherapy in treating metastatic diseases in liver remains unclear. Meanwhile, there is an unmet need to enhance the efficacy of immunotherapy in managing liver metastasis in melanoma.

Of all the ablative modalities available, cryoablation is believed to have the capability of generating the strongest antitumor immune responses [8]. In the central ablation zone of cryoablation, the temperature drops below − 40 °C and can result in direct cell death [9], leading to the release of abundant antigens and cytokine milieu that consists of IFN-γ, TNF-α, IL-2 and IL-12, which facilities Th1 immune response [10]. Cryoablation in treating liver metastasis is a safe and mature technique [11–13], and adding it to immune checkpoint inhibitors has the potential to break the anti-inflammatory microenvironment in liver and tune the balance from immune tolerance towards anti-tumor immune response.

Hepatic metastases from melanoma preferentially recruit blood supply from the arterial system [14]. Melanoma patients with disseminated disease predominantly in liver are potential candidates for hepatic arterial therapies. Past studies on pharmacokinetics showed intra-arterial hepatic administration of cytotoxic drug can offer a much higher local drug concentration than the intravenous route [15]. Hepatic arterial infusion of bevacizumab can achieve significantly more reduction in tumor volume and decreasement in cell proliferation than systemic administration of bevacizumab for colorectal liver metastasis in rat model [16].Theoretically, arterial infusion of anti-PD-1 antibody for hepatic metastases has the potential to achieve higher drug concentration for tumoral infiltrative lymphocytes (TILs) than the intravenous route, which may be translated into improved anti-tumor immune response.

**Fig. 1:**
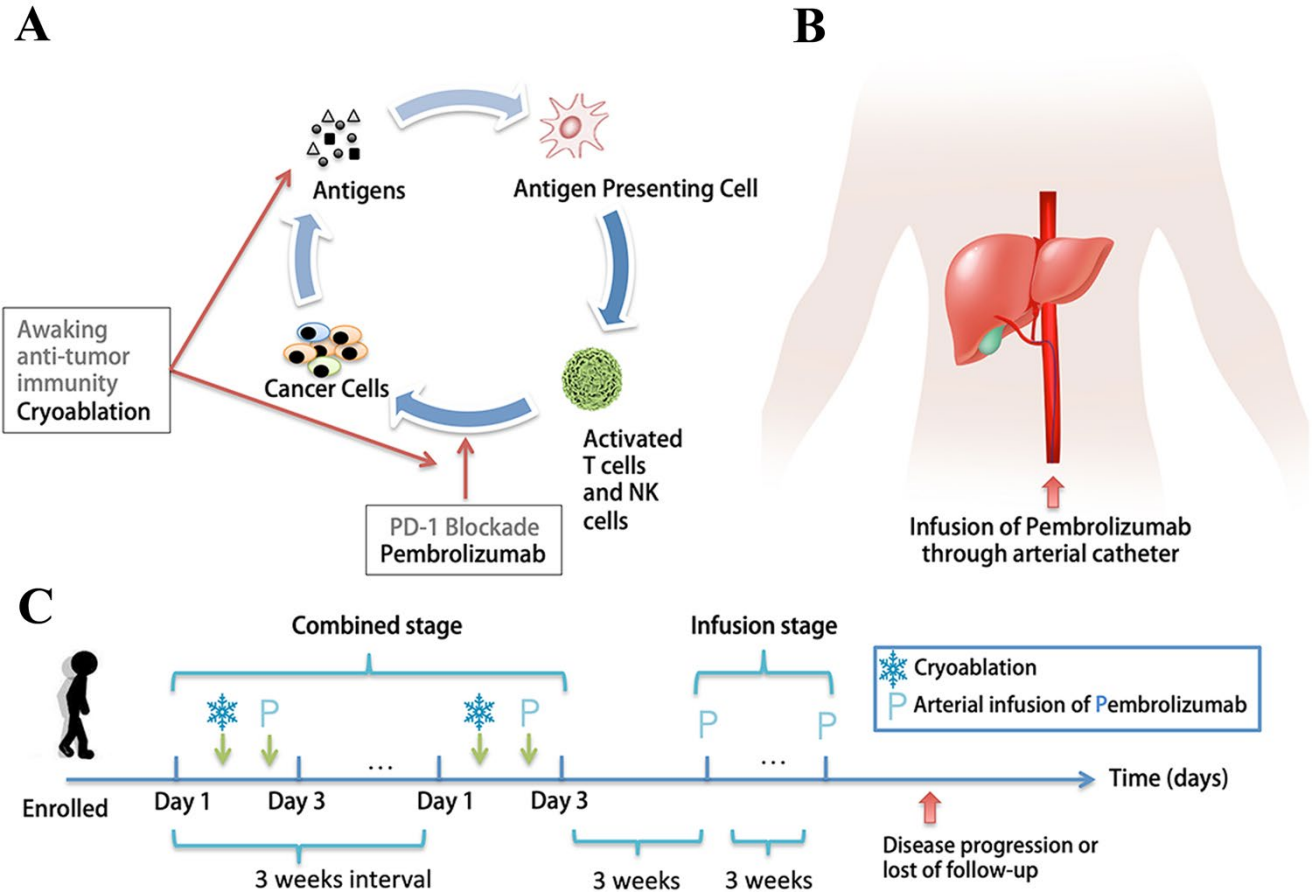
Rationale and schematic design of the combined CATAP treatment. The cryoablation of target tumor releases neoantigens and Th1 cytokines in local tumor microenvironment, facilitating the activation of T cells and NK cells; meanwhile, PD-1 blockade using pembrolizumab inhibit the PD-L1/PD1 ligation induced energy of immune cells (**a**). Schematic charts explaining the process of transarterial infusion of pembrolizumab (**b**) and the sequencing of the planned combination treatment (**c**)

Therefore, we designed a combination protocol by combining cryoablation and transarterial infusion of pembrolizumab (CATAP) to treat melanoma patients with multiple/disseminated liver metastasis (Fig. 1a, b). In this registered proof-of-concept study, we aim to report the preliminary results on efficacy and safety of this treatment strategy.

## Materials and method

### Patients

This ambispective cohort study retrospectively reviewed the medical records of a consecutive series of 15 melanoma patients with multiple hepatic metastases who received the combined therapy of cryoablation and transarterial infusion of pembrolizumab at Sun Yat-sen University Cancer Center (SYSUCC) from September 1, 2018, to May 30, 2019. The treatment decision was made together by patients, and a multidisciplinary team consists of oncologists, interventional radiologists, radiologists and surgeons. The inclusion criteria were: (a) histological diagnosis of melanoma; (b) presence of unresectable multiple/disseminated liver metastasis; (c) either failure, or patients’ refusal of first-line systemic therapies or liver-directed therapies; (d) performance score 0 or 1 based on the Eastern Cooperative Oncology Group (ECOG) Performance Scale; (e) absence of active or documented history of autoimmune diseases; (f) completed at least one cycle of treatment in the combined stage of CATAP therapy. The exclusion criteria were: (a) having received additional therapies before disease progression and (b) presence of other concurrent malignancies.

Starting from the commencement of the combination treatment, all patients were routinely assessed for safety and treatment response. From July 24, 2019, onward, a total of 11 surviving patients were prospectively followed up for evaluation of treatment outcome and toxicity; the rest four patients had either passed away or lost to follow-up. The Hospital Ethics Committee of SYSUCC approved this study (B2019-073-01), and patients with prospective follow-up signed written informed consent. The Chinese Trial Register (ChiCTR) identifier was: ChiCTR1900024899.

### Combination strategy and treatments

The CATAP treatment strategy included two stages: the combined stage and the infusion stage. In the combined stage, each patient was recommended to receive four cycles of the following combination treatment: On day 1, subtotal cryoablation was performed, in which one to two intrahepatic lesions were ablated completely while other lesions (intrahepatic and extrahepatic) were left untreated; On day 3 (within 48 h), pembrolizumab was infused transarterially. This combination treatment was repeated every three weeks, and the combined stage could be terminated based on patients’ requests, high ECOG PS score (≥ 2), due to intolerable toxicity or confirmed disease progression. After the end of combined stage, in the infusion stage, monotherapy of transarterial pembrolizumab infusion was recommended at three-week interval until disease progression, occurrence of unacceptable toxicity or lost to follow-up (Fig. 1c).

The cryoablation was performed using Visual-ICE™ System (Galil Medical, Israel). In each procedure, 1 or 2 intrahepatic lesions with largest diameter up to 5 cm were ablated; targeted lesions were chosen at the direction by interventional radiologists (W.F. and W.L. with 15 and 13 years of experience in percutaneous cryoablation, respectively) based on technical factors, such as away from major intrahepatic vessels, ease of access and treated with full ablative intent. For all procedures, local anesthesia with 2% lidocaine was performed. The cryoprobes were inserted into the targeted lesions under CT guidance, and two 10-min freezing cycles, separated by a passive 10-min and an active 2-min thawing session, were performed. During the procedure, a CT scan was performed each 3–5 min to monitor the margin of ice ball. The technical success of cryoablation was defined as the ice ball extending at least 1 cm beyond the boundaries of the tumor. A non-contrast CT scan was performed to identify any early complications after removal of cryoprobes.

Transarterial infusion of pembrolizumab was performed starting with a 3.5-French catheter inserted into the celiac trunk for arteriography. Then, a 2.7-French microcatheter was super-selectively placed into the hepatic artery proper, followed with arterial infusion of a homogeneously mixed solution of pembrolizumab (3 mg/kg) and 100 ml normal saline, which takes about 25 min (4 mL/min).

### Follow-up and endpoints assessment

Patients were followed up every 6–12 weeks with contrast-enhanced MRI/CT scan during the combination treatment. Objective response was evaluated in lesions not subject to ablation every 8–12 weeks by two experienced radiologists using Response Evaluation Criteria in Solid Tumors, version 1.1 (RECIST v1.1) [17]. The primary endpoint was objective response rate (ORR), which is defined as the percentage of patients with a best overall response of complete response (CR) or partial response (PR) based on RECIST v1.1; patients with ORR of CR or PR were defined as responders. Response assessment will be terminated if patients received treatments other than the CATAP treatment after disease progression. Secondary end points included progression-free survival (PFS), overall survival (OS) and safety profiles. The overall PFS was defined as the time from the start of combination treatment to confirmation of disease progression or death by any causes, and hepatic PFS was defined as the time from the start of combination treatment to confirmation of disease progression in liver. The OS was defined as the time from the start of combination treatment to death by any causes. Adverse events (AEs), including pre-specified immune-mediated adverse events, were collected throughout the treatment until 30 days (90 days for serious AEs) after the last infusion of pembrolizumab or before the start of a new anticancer therapy. For the retrospective portion, adverse events recorded through each outpatient and hospitalization were reviewed; for the prospective portion, adverse events were recorded through each outpatient, each hospitalization and monthly phone calls by experienced research nurse. All AEs were graded based on the NCI Common Terminology Criteria for Adverse Events v5.0.

### Immune correlative studies

Patients were recommended to conduct blood sampling 1 day prior and 1 day after each treatment of cryoablation and arterial infusion of pembrolizumab during the combination treatment. The subsets of lymphocytes were analyzed by multicolor flow cytometry. Levels of Th1 and Th2 cytokines, including IL-2, IL-4, IL-6, IL-10, TNF and IFN-γ, were assessed using immunofluorescence analysis, with a detection limit from 2.5 pg/ml to 5000 pg/ml.

### Tissue samples and next-generation sequencing

Thirteen (86.7%) of the enrolled patients had available tumor biopsy samples of liver metastases. QIAamp DNA FFPE tissue kit (Qiagen) was used to extract tissue DNA according to the manufacturer’s instructions. Qubit dsDNA assay (Life Technologies) was used to measure DNA concentration. DNA shearing was performed using Covaris M220, followed by end repair, phosphorylation and adaptor ligation. DNA fragments (200–400 bp) were selected using Agencourt AMPure beads (Beckman Coulter, Brea, CA, USA) followed by hybridization with capture probes baits, hybrid selection with magnetic beads and polymerase chain (PCR) reaction amplification. The quality and size of the fragments was assessed using bioanalyzer high-sensitivity DNA assay. Fifty nanogram DNA and twelve PCR cycles were used for library construction and amplification, respectively. Sequencing of samples was finished on Nextseq500 sequencer (Illumina, Inc., San Diego, CA, USA) with pair-end reads (read length 150 bp). A panel manufactured by Burning Rock Biotech, Guangzhou, China, was used in this study, which covers selected exons and introns of 295 cancer-related genes, spanning 2.02 MB of human genome.

### Statistical analysis and graphical visualization

Efficacy by response rate was reported as percentages. Pearson Chi-squared test was used to compare categorical variables between groups, and Fisher’s exact test was utilized when expected count of any cell in the contingency table is less than 5. Paired t test was used to test the differences of a series of paired observations on serum immune correlative studies before and after the combination treatment. Mann–Whitney U test was utilized to compare the tumor mutation burden (TMB) between responders and non-responders. All analyses were done using SPSS 21.0. The swimmer plot, spider plot and heatmap were made using R 3.6.1 (The R Foundation for Statistical Computing, 2019).

## Results

### Patient characteristics

The baseline characteristics of the enrolled patients are listed in Table 1. The median age of the population was 53 (range 32–76). Nine patients (60.0%) had primary cutaneous melanoma, and six patients (40.0%) had primary uveal melanoma. The majority of patients had metachronous liver metastases (13/15, 86.7%), high number (> 20) of intrahepatic lesions (9/15, 60.0%) and concurrent extrahepatic metastases (13/15, 86.7%). Thirteen (86.7%) patients started this combination treatment after failure of previous treatment, while two (13.3%) patients chose it as first-line treatment. The median follow-up time was 8.4 (95% CI 7.9–8.9) months.

**Table 1:** Patient baseline characteristics

Category	Whole Cohort *N*, (%)
Age, median (range)	53 (32–76)
Sex	
Male	8 (53.3)
Female	7 (46.7)
Tumor origin	
Cutaneous	9 (60.0)
Uveal	6 (40.0)
ECOG	
0	13 (86.7)
1	2 (13.3)
LDH level	
Normal	6 (40.0)
Elevated	9 (60.0)
Metastatic onset	
Synchronous	2 (13.3)
Metachronous	13 (86.7)
Number of intrahepatic metastasis	
4–20	6 (40.0)
> 20	9 (60.0)
Intrahepatic tumor size (cm)	
< 5	10 (66.7)
≥ 5	5 (33.3)
Extrahepatic metastasis	
Absent	2 (13.3)
Present	13 (86.7)
Location of extrahepatic metastasis	
Lung	7 (46.7)
Bone	8 (53.3)
Subcutaneous	4 (26.7)
Other^a^	4 (26.7)
Number of metastatic sites	
1	2 (13.3)
2	6 (40.0)
3	4 (26.7)
> 3	3 (20.0)
Previous treatments	
Pembrolizumab i.v	6 (40.0)
Chemotherapy	3 (20.0)
Targeted therapy	4 (26.7)
Embolization-based therapy	2 (13.3)
Other therapy	5 (33.3)

^a^Other locations includes pancreas, spleen, kidney, brain and retroperitoneal lymph nodes

### Treatment feasibility and safety

During the combined stage, the mean cycles of combination treatment were 2.2 (range 1–4). Technical success of cryoablation was achieved for all targeted intrahepatic lesions (51/51; 100.0%), and technical success of arterial catheter placement was achieved in 15/15 (100.0%) patients. In the fusion stage, the mean number of transarterial pembrolizumab infusions was 1.9 (range 0–8), with all procedures conducted successfully. Regarding cryoablation, no early major complications requiring transfusion or embolization occurred; late major complications including abscess, infarction, tumor seeding and biloma were not observed during follow-up**.** The most frequent treatment-related grade 1–2 AEs were fatigue (53.4%), arthralgia (33.3%), nausea (33.3%), pruritus (20.0%), and elevation of aspartate transaminase (AST) or alanine transaminase (ALT) (20.0%). No grade 3–4 AEs were observed (Table 2). No patients discontinue therapy or reduce dosage of pembrolizumab due to adverse events or toxicities.

**Table 2:** Adverse events considered to be drug related by investigators (CTCAE v.5.0)

Adverse events	Grade 1 (*n*, %)	Grade 2 (*n*, %)	Grade 3/4 (*n*, %)
Any	9 (60.0)	3 (20.0)	0 (0.0)
Fatigue	7 (46.7)	1 (6.7)	0 (0.0)
Pyrexia	1 (6.7)	0 (0.0)	0 (0.0)
Arthralgia	4 (26.7)	1 (6.7)	0 (0.0)
Myalgia	1 (6.7)	1 (6.7)	0 (0.0)
Headache	0 (0.0)	0 (0.0)	0 (0.0)
Pruritus	3 (20.0)	0 (0.0)	0 (0.0)
Rash	1 (6.7)	0 (0.0)	0 (0.0)
Nausea	5 (33.3)	0 (0.0)	0 (0.0)
Vitiligo	2 (13.3)	0 (0.0)	0 (0.0)
Diarrhea	2 (13.3)	0 (0.0)	0 (0.0)
Hypothyroidism	0 (0.0)	0 (0.0)	0 (0.0)
Adrenal insufficiency	0 (0.0)	0 (0.0)	0 (0.0)
Elevated Aspartate Aminotransferase	2 (13.3)	0 (0.0)	0 (0.0)
Elevated Alanine Aminotransferase	1 (6.7)	1 (6.7)	0 (0.0)

### Efficacy

**Fig. 2:**
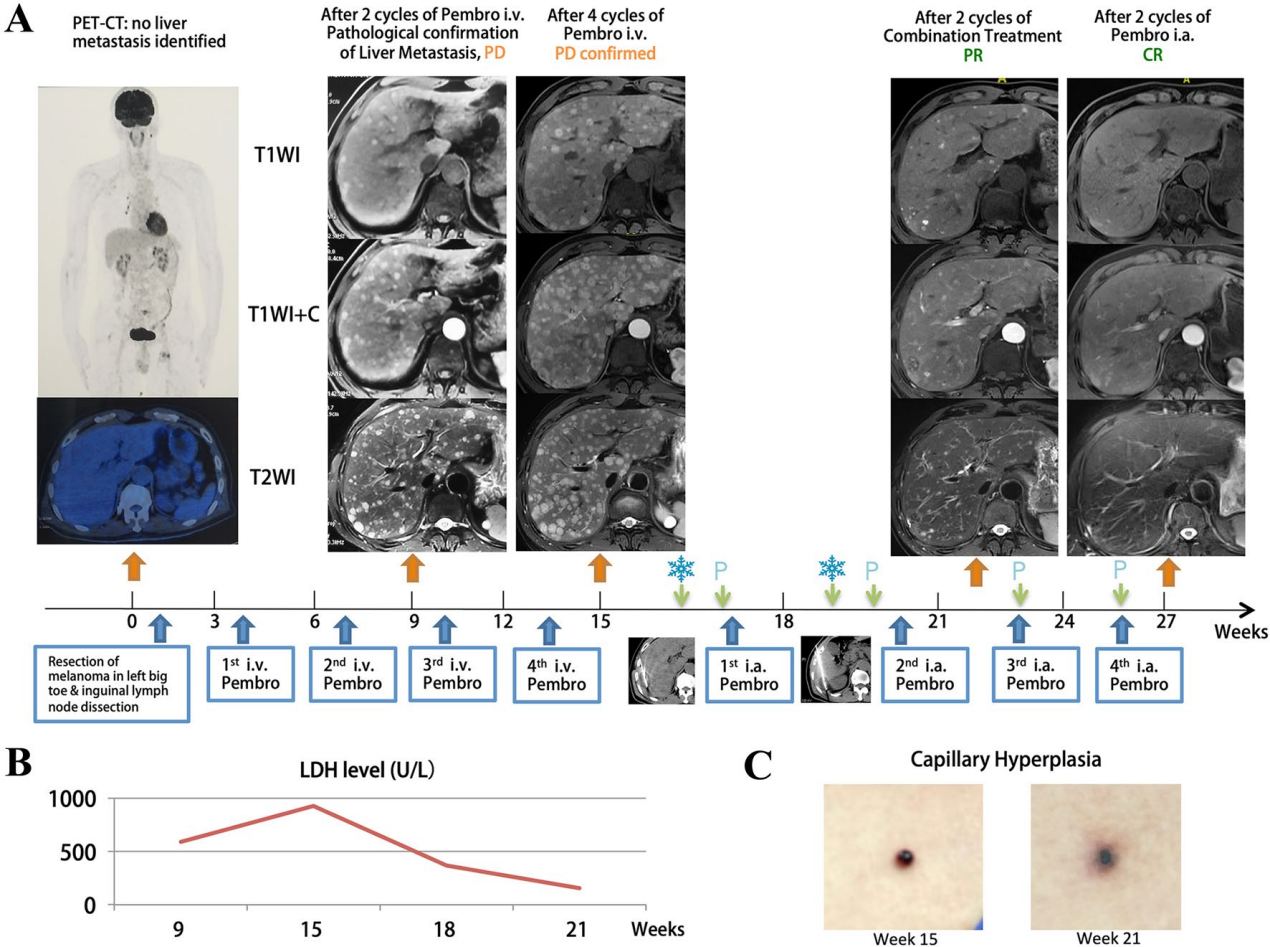
Example of a patient with complete response. (**a**) A 56-year old male with melanoma in left big toe and inguinal lymph nodes metastases underwent surgical resection and adjuvant i.v. pembrolizumab therapy. Multiple liver metastases, bony metastases and pancreatic metastases were identified after two cycles of i.v. pembrolizumab; the disease progressed after additional two cycles of i.v. pembrolizumab treatment. Seeking for novel treatment options, the patient agreed to receive the CATAP treatment. A major PR was observed after two cycles of combination treatment; CR was achieved after two more cycles of i.a. infusion of pembrolizumab. (**b**) The change of serum LDH level during the treatment. (**c**) Capillary hyperplasia on the chest after i.v. pembrolizumab turn for the better after the combined CATAP treatment

Of the 15 patients enrolled, the best treatment response included 1 (6.7%) complete response (CR), 3 (20.0%) partial response (PR), 3 (20.0%) stable disease (SD) and 8 (53.3) progression disease (PD) (Table 3; Fig. 2). The ORRs for the entire cohort and cutaneous melanoma group were 26.7% (95% CI 4.3–49.0%) and 33.3% (95% CI 2.5–64.1%), respectively. Two of six patients (33.3%) with cutaneous melanoma and failed after first-line intravenous pembrolizumab treatment responded to the CATAP treatment. No significant differences in overall response rates were detected in covariates such as tumor origin, intrahepatic tumor size and the usage of intravenous pembrolizumab before (Table 4).

**Table 3:** CATAP treatment and treatment response in enrolled cohort

Patient number	Gender	Age	Tumor origin	Pembro i.v. before	Number of intrahepatic lesions	Diameter (mm)	Extrahepatic metastasis	Cycles in combined stage	Reason for discontinuity in combined stage^a^	Cycles in infusion stage	Reason for discontinuity in infusion stage^a^	Best treatment Response
1	Female	62	Cutaneous	No	> 20	24	Present	3	Self	2	Self	Partial response
2	Male	43	Uveal	No	> 20	21	Present	1	disease progression	0	Disease progression	Progression
3	Male	53	Cutaneous	No	> 20	42	Present	3	Disease progression	1	Disease progression	Progression
4	Male	76	Cutaneous	No	4–20	55	Present	2	Self	0	Self	Stable disease
5	Female	43	Uveal	No	> 20	30	Present	3	Self	6	Self	Partial response
6	Female	68	Uveal	No	4–20	13	Absent	2	Disease progression	2	Disease progression	Progression
7	Female	32	Cutaneous	Yes	> 20	60	Present	2	Self	0	Self	Stable disease
8	Male	34	Cutaneous	Yes	4–20	13	Present	2	Self	2	Self	Partial response
9	Female	57	Uveal	No	4–20	52	Present	4	–	3	Disease progression	Stable disease
10	Female	55	Uveal	No	4–20	65	Absent	3	High PS score	2	Disease progression	Progression
11	Male	53	Cutaneous	Yes	4–20	32	Present	2	Disease progression	1	Disease progression	Progression
12	Female	53	Uveal	No	> 20	60	Present	1	High PS score	0	High PS score	Progression
13	Male	54	Cutaneous	Yes	> 20	17	Present	2	Disease progression	0	Disease progression	Progression
14	Male	33	Cutaneous	Yes	> 20	30	Present	1	Self	1	Disease progression	Progression
15	Male	56	Cutaneous	Yes	> 20	27	Present	2	Self	8	CR achieved	Complete response

^a^Self refers to discontinuity of treatment based on patients’ requests

**Table 4:** Best treatment response assessed by RECIST 1.1

Categories	CR (*n*, %)	PR (*n*, %)	SD (*n*, %)	PD (*n*, %)	ORR (*n*, %)	*P*
Whole Cohort	1 (6.7)	3 (20.0)	3 (20.0)	8 (53.3)	4 (26.7)	
Tumor origin						0.462
Cutaneous origin	1 (11.1)	2 (22.2)	2 (22.2)	4 (44.4)	3 (33.3)	
Uveal origin	0 (0.0)	1 (16.7)	1 (16.7)	4 (66.7)	1 (16.7)	
Intrahepatic tumor size (cm)						0.154
< 5	1 (10.0)	3 (30.0)	0 (0.0)	6 (60.0)	4 (40.0)	
≥ 5	0 (0.0)	0 (0.0)	3 (60.0)	2 (40.0)	0 (0.0)	
Previous Pembrolizumab i.v						0.538
No	0 (0.0)	2 (22.2)	2 (22.2)	5 (55.6)	2 (22.2)	
Yes	1 (16.7)	1 (0.0)	1 (0.0)	3 (66.7)	2 (33.3)	

ORR, overall response rate. *P* value was calculated by comparing ORR rates between subgroups using two sided Fisher-exact Chi-square test

**Fig. 3:**
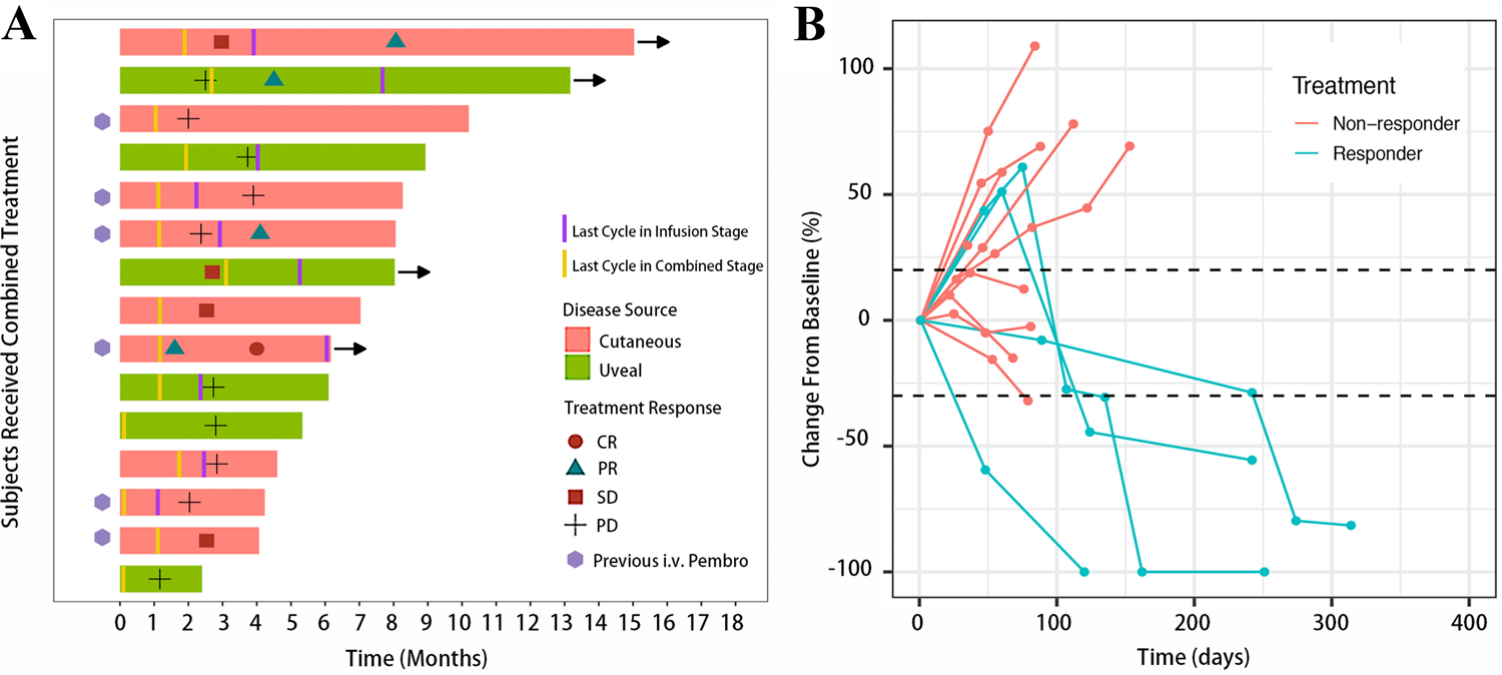
Response of enrolled population receiving CATAP treatment. (**a**) Swimmer’s plot showing patients’ time to response and current status if applicable; arrow indicates the patient still on study. (**b**) Spider plot showing the change of sum of target lesions over time based on RECIST 1.1 criteria

The median time to response was 4.3 months (Fig. 3a). All responders had achieved more than 50% decrease in the size of target lesions (Fig. 3b). No significant differences in the distribution of covariates between responders and non-responders were identified.

The median overall PFS time and hepatic PFS time in the cohort were 4.0 (95% CI 2.5–5.5) and 5.73 (95% CI 1.1–10.4) months, respectively. The estimated 6- and 12-month overall PFS rates were 40.0% and 18.2%, respectively, and the estimated 6- and 12-month hepatic PFS rates were 42.9% and 23.8%, respectively. The median OS time was not reached. The estimated 6- and 12-month OS rates were 72.4% and 61.3%, respectively.

### Immune correlatives and NGS

**Fig. 4:**
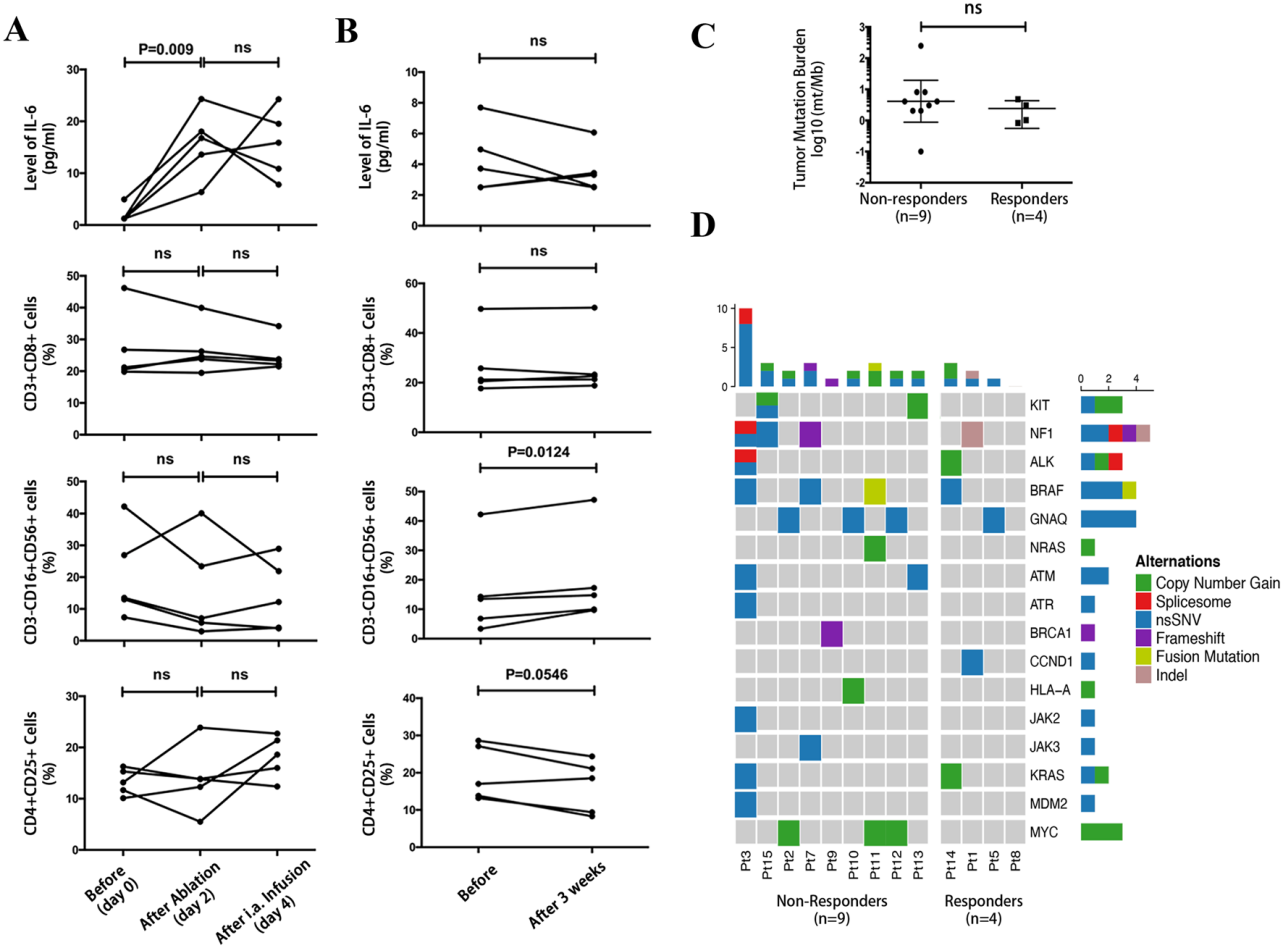
The immune correlative studies and NGS of the enrolled patients. (**a**) Dynamic changes of serum IL-6 and lymphocytes subsets of patients with paired test results during the first combined CATAP treatment of the combined stage. (**b**) Changes of serum IL-6 and lymphocytes subsets of patients with paired test results before and 3 weeks after the first combined CATAP treatment of the combined stage. (**c**) Tumor mutation burden of responders and non-responders. (**d**) Heatmap of genetic alterations in pretreatment tumors of responding and non-responding patients. Melanoma signature genes, PD-1 blockade-associated genes that found mutated in our cohort and mutated genes occurred in more than 25% in non-responding patients while absent in the responding group were displayed

In the combined stage, the change in serum cytokines and subsets of lymphocytes during the first combination treatment was obtained in 5 (33.3%) patients (Fig. 4a). The serum level of IL-6 immediately increased after cryoablation and maintains stable during the arterial infusion of pembrolizumab. On the other hand, no significant changes in the subsets of lymphocytes during the first combination treatment were found. The serum level of IL-2, IL-4, IL-10, TNF and IFN-γ in most patients was below the detection limit (< 0.25 pg/ml), and therefore, no significant changes could be detected. Five patients (33.3%) with immune correlative tests before and three weeks after the first combination treatment were also analyzed (Fig. 4b). No significant change in the serum level of IL-6 and subsets of CD3 + CD8 + lymphocytes was observed while it was interesting to note that the proportion of CD3-CD16 + CD56 + cells (NK cell) significantly increased (*P* = 0.0124) and a marginal significant decrease in CD4 + CD25 + cells (Treg; *P* = 0.0546) were identified three weeks after.

Of the patients with data on NGS, 9 were non-responders (69.2%) and 4 (30.8%) were responders. All patients had microsatellite stable (MSS) tumors. No significant difference in tumor mutation burden was found between the two groups (Fig. 4c). Based on the results of 295 cancer-related gene panel, the mutations of melanoma signature genes and PD-1 blockade-associated genes are displayed in Fig. 4d. One patient contained mutation in JAK2 was non-responder. No patients had MDM2 amplification, PTEN, BRCA2 or JAK1 mutation. About 33.3% non-responders contained MYC copy number gain, while no responders had this genetic alteration. Further deductions could not be made due to limited sample size.

## Discussion

This ambispective cohort study demonstrated a proof of concept that the designed treatment protocol of cryoablation combined with transarterial PD-1 blockade therapy (CATAP) is safe and can achieve antitumor immune response for liver metastasis of melanoma.

Although significant improvement in the management of metastatic melanoma is achieved with the advent of immune checkpoint inhibitors, the prognosis of cutaneous or uveal melanoma with bulky liver metastases remains poor and novel therapeutics are needed [18]. Wen et al. retrospectively analyzed 52 Chinese patients with metastatic melanoma receiving immune checkpoint inhibitors and found that pembrolizumab-containing therapy was most effective for lung, lymph node or subcutaneous lesions. The response rate of pembrolizumab-containing therapy in patients with or without liver metastasis was 0% (0/14) and 37.5% (9/24) respectively [19]. Tumeh et al. analyzed the combined data of KEYNOTE-002 and KEYNOTE-006 and found that the response rate of pembrolizumab monotherapy in patients with liver metastasis was 14.7% (5/34), with a median PFS time of 2.7 months [4]. Although combined nivolumab and ipilimumab had been demonstrated to have a numerically higher response rate and longer PFS than PD-1 blockade alone, its efficacy in liver metastases had not been reported [20, 21]. In the present study, the response rate of the designed CATAP treatment protocol in the entire cohort was 26.7%, which was higher than previously reported data. It is notable that previous studies demonstrated that immune checkpoint inhibitors, either targeting PD-1 or cytotoxic T-lymphocyte antigen-4 (CTLA-4), have generally low response rates (2–5%) in metastatic UM patients [18, 22]. Based on the results of a recently published trial, rapid progression was observed in all UM patients with extensive liver metastases after anti-PD-1-based therapy [23]. In our study, one in six (16.7%) UM patients responded to CATAP therapy. These results indicated that there is a response signal for CATAP treatment in this group of patients, which merits further evaluation in a phase 2 randomized trial against intravenous pembrolizumab treatment.

In KEYNOTE-006, no patients with progression of hepatic lesions after first course of pembrolizumab responded to second course of pembrolizumab [1]. In our study, one patient even achieved CR through CATAP therapy after failure of intravenous pembrolizumab treatment. The possible explanation for this phenomenon may be that PD-1 blockade monotherapy failed to facilitate the identification of tumor neoantigens, while cryoablation of the intrahepatic lesions could be viewed as a vaccine conferring “auto-vaccination” [24]. The preserved cryoablated tumor neoantigens could be presented and processed by the host’s immune system, thus resulting in a more robust anti-tumor immune response [10]. Meanwhile, transarterial infusion of pembrolizumab into the tumor feeding artery might provide faster and more complete blockade of PD-1 on tumor infiltrative immune cells as compared to the intravenous route, facilitating anti-tumor immune response. This case provided us with an insight that compared with the intravenous pembrolizumab monotherapy, CATAP therapy might be able to tilt the body's immune balance toward enhanced anticancer immune response. This effect might also be reflected by the results on increased NK cells and decreased Tregs in peripheral blood after single cycle of treatment in the combined stage, as both increased NK cells [25, 26] and decreased Tregs [27, 28] had been reported to correlate with favorable response of immunotherapy in melanoma.

Although no significant correlations between clinical factors and treatment response were identified, all responders had intrahepatic tumors with maximal diameter less than 5 cm, indicating intrahepatic tumor size could be a potential predictor of response for CATAP therapy. Joseph et al. calculate the baseline tumor size (BTS) by adding the sum of the longest dimensions of all measurable baseline target lesions and evaluate its correlation with efficacy of pembrolizumab in 583 metastatic melanoma patients of KEYNOTE-001 study. Their study results showed BTS is an independent prognostic marker of OS (*P* < 0.001) but not ORR [29]. Currently, there is still no valid evidence supporting tumor size as a predictive factor for immunotherapy. The observed potential correlation between intrahepatic tumor size and response for CATAP therapy needed to be validated in a larger prospective cohort in the future.

Certain genetic mutations in melanoma, including JAK1, JAK2, B2M and PTEN, etc., correlate with negative treatment response of PD-1 blockade therapy, while BRCA2 mutations were reported enriched in melanomas responsive to this treatment [30, 31]. In our series, one patient with JAK2 mutation was non-responder, which is consistent with previous studies. The predictive value of tumor mutation burden had been confirmed in specific cancer types, including melanoma [32, 33]. In Forschner’s study, a cutoff value of 23.1 Mut/Mb was utilized to separate the TMB of metastatic melanoma patients into TMB low or intermediate and TMB high, which was found effective in predicting the response and overall survival under combined CTLA-4 and PD-1 antibody therapy. However, in our series, only one patient (non-responder) had TMB > 23.1 Mut/Mb. We used nonparametric test to compare the tumor mutation burden between the two groups (responders vs. non-responders), and no significant difference was found. Further studies are warranted to identify predictors of CATAP treatment for liver metastatic melanoma.

In KEYNOTE-006, it is reported that grade 3–5 AEs occurred in 17% of patients receiving pembrolizumab, and the most common AEs were diarrhea (1–3%), colitis (1.4–2.5%) and hepatitis (1.1–1.8%) [34]. On the other hand, grade 3–5 adverse events occurred in 20.0–27.3% of patients receiving ipilimumab monotherapy. The combination of pembrolizumab and ipilimumab in melanoma patients could raise the rate of grade 3–4 AEs to about 55% [21]. In this study, no grade 3–4 AEs related to CATAP therapy were identified. Previous studies reported that cryoshock phenomenon may happen in patients receiving cryoablation for liver metastasis, with an incidence rate of about 1% [35]. In our study cohort, no cryoshock phenomenon was observed in patients received CATAP therapy. These results suggested that this combined approach was safe and tolerable.

The current study had several limitations. Firstly, this was a retrospective and prospective data collection study, and the nature of retrospective study design may lead to potential patient selection bias. Secondly, the enrolled sample size was relatively small, and a multicenter randomized controlled trial comparing the CATAP treatment and intravenous pembrolizumab monotherapy is needed to confirm the efficacy of this novel therapy.

## Conclusion

Cryoablation combined with transarterial infusion of pembrolizumab (CATAP) treatment is safe, and the CATAP treatment protocol designed in this study has promising clinical activity in the management of liver metastasis of melanoma. A larger prospective study is needed to confirm its efficacy in the future.

## Abbreviations

AEs: Adverse events
AST: Aspartate transaminase
ALT: Alanine transaminase
CATAP: Combined cryoablation and transarterial infusion of pembrolizumab treatment strategy
CTLA-4: Cytotoxic T-lymphocyte antigen-4
ECOG: Eastern cooperative oncology group
LSECs: Liver sinusoidal endothelial cells
PD-1: Programmed cell death protein 1
TILs: Tumoral infiltrative lymphocytes
TMB: Tumor mutation burden

## References

[1] Robert C, Ribas A, Schachter J (2019). Pembrolizumab versus ipilimumab in advanced melanoma (KEYNOTE-006): post-hoc 5-year results from an open-label, multicentre, randomised, controlled, phase 3 study. The Lancet Oncol.

[2] Rodriguez-Cerdeira C, Carnero Gregorio M, Lopez-Barcenas A, Sanchez-Blanco E, Sanchez-Blanco B, Fabbrocini G, Bardhi B, Sinani A, Guzman RA (2017). Advances in immunotherapy for melanoma: a comprehensive review. Mediators Inflamm.

[3] Robert C, Ribas A, Wolchok JD (2014). Anti-programmed-death-receptor-1 treatment with pembrolizumab in ipilimumab-refractory advanced melanoma: a randomised dose-comparison cohort of a phase 1 trial. Lancet.

[4] Tumeh PC, Hellmann MD, Hamid O (2017). Liver metastasis and treatment outcome with Anti-PD-1 monoclonal antibody in patients with melanoma and NSCLC. Cancer Immunol Res.

[5] Knolle P, Schlaak J, Uhrig A, Kempf P, Meyer zum Buschenfelde KH, Gerken G (1995). Human Kupffer cells secrete IL-10 in response to lipopolysaccharide (LPS) challenge. J Hepatol.

[6] Jenne CN, Kubes P (2013). Immune surveillance by the liver. Nat Immunol.

[7] John B, Crispe IN (2004). Passive and active mechanisms trap activated CD8+ T cells in the liver. J Immunol.

[8] Almeida JP, Drezek RA, Foster AE (2014). Controlling melanoma at local and systemic levels: is a combination of ablative therapy and immunotherapy the way forward?. Immunotherapy.

[9] Chu KF, Dupuy DE (2014). Thermal ablation of tumours: biological mechanisms and advances in therapy. Nat Rev Cancer.

[10] Abdo J, Cornell DL, Mittal SK, Agrawal DK (2018). Immunotherapy plus cryotherapy: potential augmented abscopal effect for advanced cancers. Front Oncol.

[11] Zhang W, Yu H, Guo Z, Li B, Si T, Yang X, Wang H (2014). Percutaneous cryoablation of liver metastases from breast cancer: initial experience in 17 patients. Clin Radiol.

[12] Kim GM, Won JY, Kim MD (2016). Cryoablation of hepatocellular carcinoma with high-risk for percutaneous ablation: safety and efficacy. Cardiovasc Interv Radiol.

[13] Xin'an L, Jianying Z, Lizhi N, Fei Y, Xiaohua W, Jibing C, Jialiang L, Kecheng X (2013). Alleviating the pain of unresectable hepatic tumors by percutaneous cryoablation: experience in 73 patients. Cryobiology.

[14] Lodh S, Maher R, Guminski A (2014). Intra-arterial infusion and chemo-embolization for melanoma liver metastases. J Surg Oncol.

[15] Munck JN, Riggi M, Rougier P (1993). Pharmacokinetic and pharmacodynamic advantages of pirarubicin over adriamycin after intraarterial hepatic administration in the rabbit VX2 tumor model. Cancer Res.

[16] Sperling J, Schafer T, Ziemann C, Benz-Weiber A, Kollmar O, Schilling MK, Menger MD (2012). Hepatic arterial infusion of bevacizumab in combination with oxaliplatin reduces tumor growth in a rat model of colorectal liver metastases. Clin Exp Metastasis.

[17] Eisenhauer EA, Therasse P, Bogaerts J (2009). New response evaluation criteria in solid tumours: revised RECIST guideline (version 1.1). Eur J Cancer.

[18] Algazi AP, Tsai KK, Shoushtari AN (2016). Clinical outcomes in metastatic uveal melanoma treated with PD-1 and PD-L1 antibodies. Cancer.

[19] Wen X, Ding Y, Li J (2017). The experience of immune checkpoint inhibitors in Chinese patients with metastatic melanoma: a retrospective case series. Cancer Immunol Immunother CII.

[20] Wolchok JD, Chiarion-Sileni V, Gonzalez R (2017). overall survival with combined nivolumab and ipilimumab in advanced melanoma. N Engl J Med.

[21] Larkin J, Chiarion-Sileni V, Gonzalez R (2015). combined nivolumab and ipilimumab or monotherapy in untreated melanoma. N Engl J Med.

[22] Luke JJ, Callahan MK, Postow MA (2013). Clinical activity of ipilimumab for metastatic uveal melanoma: a retrospective review of the Dana-Farber Cancer Institute, Massachusetts General Hospital, Memorial Sloan-Kettering Cancer Center, and University Hospital of Lausanne experience. Cancer.

[23] Johnson DB, Bao R, Ancell KK, Daniels AB, Wallace D, Sosman JA, Luke JJ (2019). Response to anti–PD-1 in uveal melanoma without high-volume liver metastasis. J Natl Compr Cancer Netw.

[24] Bastianpillai C, Petrides N, Shah T, Guillaumier S, Ahmed HU, Arya M (2015). Harnessing the immunomodulatory effect of thermal and non-thermal ablative therapies for cancer treatment. Tumour Biol.

[25] Lee H, Quek C, Silva I (2019). Integrated molecular and immunophenotypic analysis of NK cells in anti–PD-1 treated metastatic melanoma patients. Oncoimmunology.

[26] Barry KC, Hsu J, Broz ML (2018). A natural killer-dendritic cell axis defines checkpoint therapy-responsive tumor microenvironments. Nat Med.

[27] Gambichler T, Schroter U, Hoxtermann S, Susok L, Stockfleth E, Becker JC (2019). Decline of PD-1 positive circulating T regulatory cells predicting more favorable clinical outcome of melanoma patients under immune checkpoint blockade. Br J Dermatol.

[28] Gambichler T, Schroter U, Hoxtermann S, Susok L, Stockfleth E, Becker JC (2019). A brief communication on circulating PD-1-positive T-regulatory lymphocytes in melanoma patients undergoing adjuvant immunotherapy with nivolumab. J Immunother.

[29] Joseph RW, Elassaiss-Schaap J, Kefford R (2018). Baseline tumor size is an independent prognostic factor for overall survival in patients with melanoma treated with pembrolizumab. Clin Cancer Res.

[30] Zaretsky JM, Garcia-Diaz A, Shin DS (2016). Mutations associated with acquired resistance to PD-1 blockade in melanoma. N Engl J Med.

[31] Hugo W, Zaretsky JM, Sun L (2017). Genomic and transcriptomic features of response to anti–PD-1 therapy in metastatic melanoma. Cell.

[32] Forschner A, Battke F, Hadaschik D (2019). Tumor mutation burden and circulating tumor DNA in combined CTLA-4 and PD-1 antibody therapy in metastatic melanoma—results of a prospective biomarker study. J Immunother Cancer.

[33] Seto T, Sam D, Pan M (2019). Mechanisms of primary and secondary resistance to immune checkpoint inhibitors in cancer. Med Sci (Basel).

[34] Robert C, Schachter J, Long GV (2015). Pembrolizumab versus ipilimumab in advanced melanoma. N Engl J Med.

[35] Ng KK, Lam CM, Poon RT, Ai V, Tso WK, Fan ST (2003). Thermal ablative therapy for malignant liver tumors: a critical appraisal. J Gastroenterol Hepatol.

